# The Bone Marrow-Mediated Protection of Myeloproliferative Neoplastic Cells to Vorinostat and Ruxolitinib Relies on the Activation of JNK and PI3K Signalling Pathways

**DOI:** 10.1371/journal.pone.0143897

**Published:** 2015-12-01

**Authors:** Bruno A. Cardoso, Hélio Belo, João T. Barata, António M. Almeida

**Affiliations:** 1 Unidade de Investigação em Patobiologia Molecular, Instituto Português de Oncologia de Lisboa—Francisco Gentil, E.P.E., Lisbon, Portugal; 2 Centro de Estudos de Doenças Crónicas, CEDOC, NOVA Medical School/Faculdade de Ciências Médicas, Universidade Nova de Lisboa, Lisbon, Portugal; 3 Instituto de Medicina Molecular, Faculdade de Medicina da Universidade de Lisboa, Lisbon, Portugal; China Medical University, TAIWAN

## Abstract

The classical BCR-ABL-negative Myeloproliferative Neoplasms (MPN) are a group of heterogeneous haematological diseases characterized by constitutive JAK-STAT pathway activation. Targeted therapy with Ruxolitinib, a JAK1/2-specific inhibitor, achieves symptomatic improvement but does not eliminate the neoplastic clone. Similar effects are seen with histone deacetylase inhibitors (HDACi), albeit with poorer tolerance. Here, we show that bone marrow (BM) stromal cells (HS-5) protected MPN-derived cell lines (SET-2; HEL and UKE-1) and MPN patient-derived BM cells from the cytotoxic effects of Ruxolitinib and the HDACi Vorinostat. This protective effect was mediated, at least in part, by the secretion of soluble factors from the BM stroma. In addition, it correlated with the activation of signalling pathways important for cellular homeostasis, such as JAK-STAT, PI3K, JNK, MEK-ERK and NF-κB. Importantly, the pharmacological inhibition of JNK and PI3K pathways completely abrogated the BM protective effect on MPN cell lines and MPN patient samples. Our findings shed light on mechanisms of tumour survival and may indicate novel therapeutic approaches for the treatment of MPN.

## Introduction

The classical BCR-ABL-negative myeloproliferative neoplasms (MPN) include Polycythaemia Vera (PV), Essential Thrombocytosis (ET) and Primary Myelofibrosis (PMF). These conditions arise from a clonal defect on myeloid progenitor cells that lead to increased proliferation of erythroid and megakaryocytic precursors resulting in the excessive production of mature blood components [1, 2]. The major clinical complications associated with these disorders are thrombohemorrhagic events, hypercatabolic state, splenomegaly, and transformation to Acute Myeloid Leukaemia (AML) [3].

The common mechanism for the three conditions is a dysregulated hyperactivity of the tyrosine kinase JAK2. The commonest cause of this is a gain of function mutation resulting in a Valine to Phenilanine substitution at the codon 617 (*JAK2V617F*) [4–7] leading to the constitutive phosphorylation of this protein with subsequent activation of several downstream signalling pathways like JAK-STAT, MAPK-RAS and PI3K [8]. The *JAK2V617F* mutation occurs in the vast majority of PV patients (up to 97%) and in a large proportion of ET and PMF patients (50–60%). In addition to this and other JAK2-activating mutations, mutations in genes encoding epigenetic modulators such as *TET2*, *ASXL1*, *EZH2* and *IDH1/2* have been described in MPN [8–10].

The molecular characterization of MPN has led to the use of JAK and HDAC inhibitors in these patients [11–15]. Ruxolitinib is a JAK1/2 inhibitor approved for the treatment of PMF and PV [11, 12, 16, 17]. The treatment of PV and PMF patients with this agent in the context of clinical trials showed significant improvement in symptoms and splenomegaly but fail to consistently eradicate the neoplastic clone [11, 12, 17].

Vorinostat (Suberoylanilide Hydroxamic Acid) is an HDAC inhibitor which has been shown to decrease cellular viability and proliferation of MPN cells *in vitro*. In mouse models of MPN, Vorinostat produced haematological responses and, reduced tumour burden [18]. The same effects were seen in in clinical trials but the tolerability was poor [14]. Other HDAC inhibitors, like Panobinostat and Givinostat, have produced disappointing results in clinical trials [13, 15].

Leukaemic cells are not isolated entities and interact with the surrounding microenvironment which provides the stimuli which allow neoplastic cells to over-compete their normal counterparts leading to their growth and progression [19, 20]. The bone marrow (BM) is a specialized organ where normal haematopoiesis takes place, but it also acts as a sanctuary in which malignant cells from a variety of haematological disorders thrive, survive and proliferate [21]. The supportive effect of the BM is mediated by the secretion soluble factors, like cytokines, but also through direct cellular contact between the leukaemic cells and the stromal marrow cells [20, 22]. In fact, the BM stroma has been implicated in the cytoprotection of leukaemic cells to a variety of pharmacological compounds [23–26]. In the context of MPN it has been shown that the BM microenvironment protected MPN cells from the cytotoxic action of the JAK inhibitor Atiprimod [27].

We investigated the protective effect that BM stroma may exert of MPN cells and the mechanism by which this effect may be exerted. Our results demonstrate that the incubation of MPN cells with BM derived stroma impairs the cytotoxic action of both Vorinostat and Ruxolitinib. This effect is achieved by the activation of survival pathways like JNK and PI3K. Importantly, the pharmacological inhibition of such signalling pathways completely revert the BM protective effect on MPN cells. These results confirm that the BM microenvironment protects MPN cells from targeted therapies and also provide a potential therapeutic strategy to overcome this protective effect.

## Material and Methods

### Primary patient samples and cell lines

MPN patient BM samples were obtained at the Haematology service of the “Instituto Português de Oncologia de Lisboa–Francisco Gentil E.P.E.” in the course of routine clinical investigations and following written informed consent. Ethics approval was obtained and all samples were treated anonymously in accordance with the Declaration of Helsinki. MPN patient characteristics and information (diagnosis; gender; age; presence of *JAK2V617F* mutation and *in vitro* response to inhibitors) are summarized in Table 1. Mononuclear cells from BM samples were separated by density gradient centrifugation and CD34^+^ cells isolated using Diamond CD34 isolation kit (Miltenyi Biotec) according to the manufacturer’s instructions. The isolated cells were cultured in IMDM medium (Sigma-Aldrich) supplemented with 20% fetal bovine serum (FBS) (Life Technologies), Antibiotics (Lonza) and L-Glutamine (Life Technologies).

**Table 1:** MPN Patient characteristics.

Patient	Diagnosis	Gender	Age at diagnosis	*JAK2V617F* mutation	Response to Vorinostat	Response to Ruxolitinib
1	AML (Post-PV)	F	64	positive	**++**	**++**
2	PV	F	60	positive	**++**	n.d.
3	AML	F	55	negative	**+++**	**++**
4	ET	M	38	negative	**+**	**+**
5	MPN-U	M	45	negative	**+**	**-**
6	ET	F	72	negative	**+++**	**+**
7	AML (Post-PV)	M	76	positive	**++**	**+**
8	MPN-CEL/HES	F	23	negative	**++**	**+**
9	PV	F	52	negative	**++**	**-**

**AML**–Acute Myeloid Leukaemia; **PV**–Polycythaemia Vera; **ET**–Essential Thrombocytosis; **MPN-U**—Myeloproliferative Neoplasm–Unclassified; **MPN-CEL/HES**—Myeloproliferative Neoplasm with chronic eosinophilic leukemia/hypereosinophilic syndrome; **F**–Female; **M**–Male; **n.d.**–not determined; **Response to Vorinostat and to Ruxolitinib–**these represent the Viability Index (VI) of Lin^-^CD34^+^ patients cells treated with Vorinostat and Ruxolitinib without stroma (- →1.00>VI>0.91; **+** → 0.90>VI>0.51; **++** → 0.50>VI>0.21 and **+++** → 0.20>VI>0.00).

All cell lines were cultured according to standard protocols. The MPN cell lines used in our study (SET-2, HEL, UKE-1) arbor the *JAK2V617F* mutation, both HEL and UKE-1 are homozygous for this mutation, while SET-2 cell line is heterozygous [28]. The MPN cell line HEL was purchased from ATCC, while SET-2 [29] and UKE-1 [30] were kindly donated by Prof. Jean Luc-Villeval. The human BM stromal cell lines HS-5 (purchased from ATCC) was kindly donated by Prof. Paolo Gia and KM-102 by Prof. Motoo Kitagawa [31].

### Production of HS-5 conditioned media

HS-5 cells were plated in T75 flasks with 15ml DMEM-10 medium (DMEM supplemented with 10% FBS, Antibiotics and L-Glutamine) (Life Technologies). Once the cells reached 70% confluence, the medium was harvested, the cells washed once and 10ml DMEM-10 medium added to the flasks. The HS-5 conditioned media was collected every 3 days of culture for a period of 9 days. Following collection, the medium was centrifuged and the supernatant was stored at -20°C.

### *In vitro* co-culture assays

HS-5 and KM-102 cell lines were cultured to 70% confluence and the MPN cells added to the stromal layer of HS-5 (+ HS-5) or KM-102 (+ KM-102) at 0.1x10^6^ cells/ml in the appropriate culture medium, either directly (for cell to cell contact) or indirectly (separated by a 0.4-mm-thick micropore membranes +HS-5 TW). In addition, MPN cells were incubated without any stromal support (no stroma) or with the HS-5 conditioned media (+ CM), diluted 50% in the respective culture media. Vorinostat (Selleckchem), Ruxolitinib (Axon Medchem), SP600125 (JNK inhibitor) (Selleckchem) and LY294002 (PI3K inhibitor) (Cayman Chemicals) were added to the co-cultures once the MPN cells adhered to the BM stroma. At the indicated time points the cells were harvested and assessed as described below for viability, gene expression and immunoblotting.

### Cellular viability assays

Harvested cells were stained with CD45-APC (Biolegend), Annexin-V- FITC (Biolegend) and Propidium Iodide (PI) (Sigma-Aldrich). Flow cytometric data was acquired on a FACSCalibur flow cytometer (BD Biosciences) and the percentage of viable cells was described as those that did not stain for Annexin-V nor PI. Viability was also assessed by PI exclusion analysis by Flow cytometry. The data was analyzed using Flow Jo software version X.0.7 (Tree Star inc.).

### Drug titration and interaction analysis

SET-2 cells were cultured alone and with HS-5 cells for 72h and viability analyzed as described above. Drugs were added to these cultures in the concentrations described in S1 Table, alone and in the following combinations: Vorinostat (#1) with JNKi-SP600125 (#3); Vorinostat (#1) with PI3Ki-LY294002 (#4); Ruxolitinib (#2) with JNKi-SP600125 (#3) and Ruxolitinib (#2) with PI3Ki-LY294002 (#4). The half maximal effective concentration (EC50) calculation was performed using the GraphPad Prism version 5.00 for Windows (GraphPad Software) and the drug interactions were calculated using the Chou-Talalay method [32].

### RNA extraction, RT-PCR and quantitative Real-Time-PCR

RNA was extracted as described earlier [33], cDNA synthesized and gene expression evaluated by quantitative Real-Time-PCR (qPCR) and normalized to the expression levels of *HPRT1* gene. Primers used for the qPCR are indicated in S2 Table. The reagents were combined according to standard protocols and the amplifications performed in a LightCycler 480 II thermocycler (Roche).

### Immunobloting

Cell lysates were prepared as described [34] and equal amounts of protein were analyzed by 10% SDS-PAGE, transferred onto nitrocellulose membranes, and immunoblotted with the antibodies described in S3 Table.

### Statistical analysis

Differences between populations were calculated using unpaired 2-tailed Student’s t test or One-way ANOVA, when appropriate using the GraphPad Prism version 5.00 for Windows (GraphPad Software). A p-value <0.05 was considered significant.

## Results

### The BM microenvironment protects MPN cells from drug-induced apoptosis

**Fig 1:**
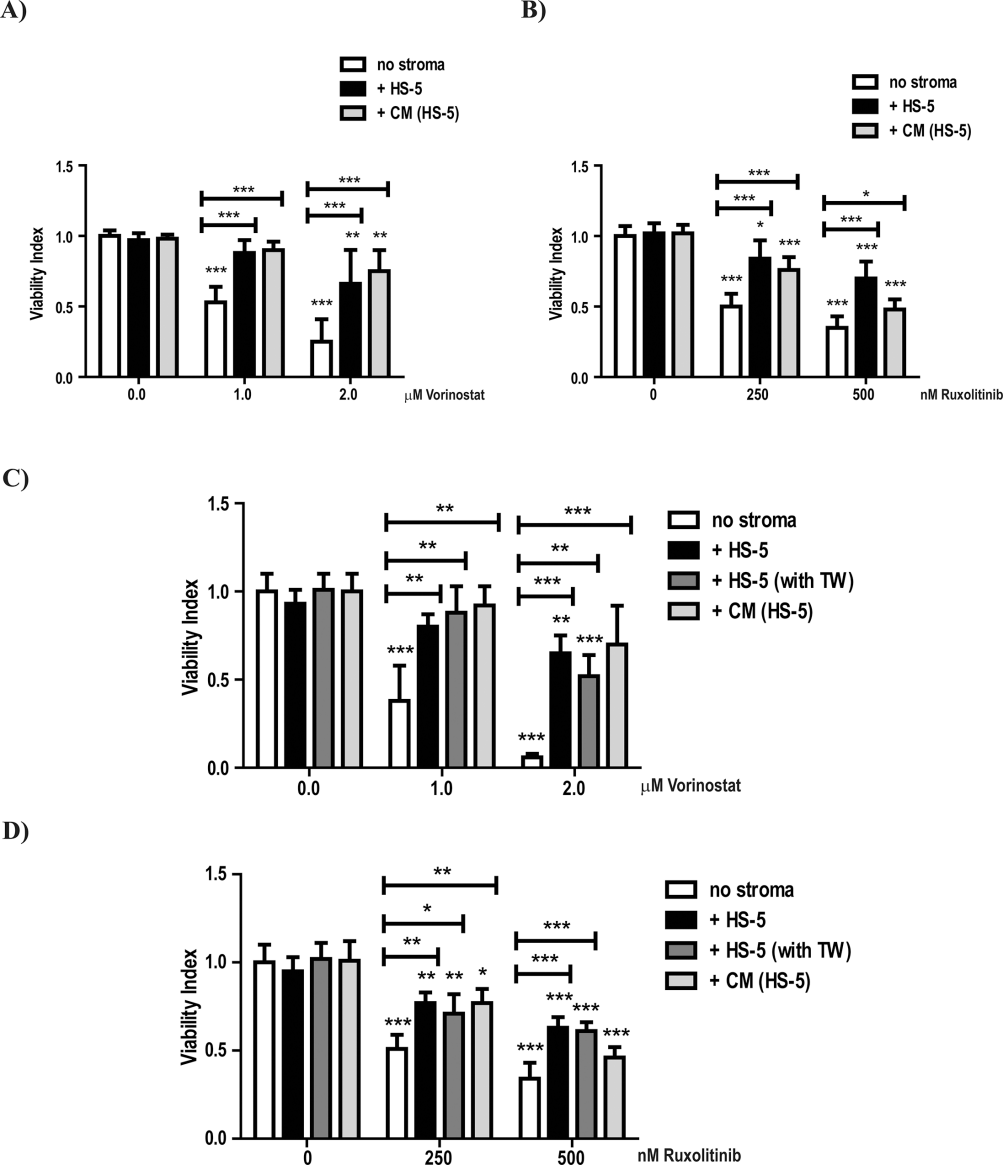
HS-5 stromal cells protect SET-2 cells from Vorinostat and Ruxolitinib- induced apoptosis. SET-2 cells were cultured *in vitro* (no stroma) and co-cultured with a stromal layer of HS-5 cells (+ HS-5), HS-5 conditioned media [+ CM (HS-5)] and or separated by a 0.4-μm-thick micropore membranes [+ HS-5 (+ TW)]. SET-2 cells were incubated in these conditions for 72h and treated with the indicated concentrations of Vorinostat (A and C) and Ruxolitinib (B and D). At 72h of co-culture, SET-2 cells were harvested, stained with CD45 (to distinguish between SET-2 and the stromal cell lines) and Annexin-V/PI (A and B) and PI alone (C and D) to determine cellular viability by Flow Cytometry analysis. The graphs indicate Viability Index that normalizes the viability values to those of the control conditions (A and C—0.0μM Vorinostat and B and D—0nM Ruxolitinib). Values indicate the mean ± standard deviation of the eight (A and B) and three (C and D) experiments performed (* 0.05>p; ** 0.01>p; *** 0.001>p).

To investigate the protective effect of the BM stroma on MPN cells we used a co-culture assay where HS-5 cells [35] were incubated with MPN cell lines and primary MPN cells. Both Vorinostat and Ruxolitinib induced apoptosis of SET-2 cells (Fig 1A and 1B—no stroma panels), which is in agreement with the reported effects of these agents in MPN cells [18, 36]. Co-culturing SET-2 cells with HS-5 cells (+ HS-5) significantly reduced apoptosis (Fig 1A and 1B—+ HS-5 panels). This protective effect of the HS-5 BM stroma was maintained for up to 6 days of co-culture (S1 Fig).

Despite protecting from apoptosis, co-culturing SET-2 cells with HS-5 did not abrogate the reported effects that Vorinostat has on gene expression, as shown by the up-regulation of HDACi transcriptional targets like *CDKN1A* [34], *IER3* [33] and *BIRC3* [37] (S2A Fig), nor the inhibitory effects of Ruxolitinib on the expression of known JAK-STAT pathway transcriptional targets, such as *BCL2* [38], *OSM* [39] and *PIM1* [40] (S2B Fig), which indicates that both Vorinostat and Ruxolitinib are being effective. Similar protective effects on apoptosis were seen in the other MPN derived cell lines HEL (S3A Fig) and UKE-1 (S3B Fig). However, unlike previous descriptions [41] we did not observe apoptosis induced by Ruxolitinib (a JAK1/2 inhibitor) in UKE-1 cells (S3B Fig). Furthermore, co-culture experiments using another BM stromal cell line (KM-102) [31] confirmed the stromal cells’ protective effect on apoptosis of SET-2 cells induced by Vorinostat (S4A Fig) and Ruxolitinib (S4B Fig). These results demonstrate that BM stromal cells maintain MPN cellular viability in the presence of both Vorinostat and Ruxolitinib.

### The protective effect of BM microenvironment is exerted through secreted factors

In order to investigate whether the protective effect of BM stromal cells requires direct cell contact (juxtacrine) or is mediated by secreted molecules (paracrine), we cultured SET-2 cells with the HS-5 conditioned media or with the HS-5 cells without direct contact between stromal and MPN cells. The conditioned media (+ CM) displayed a similar protective effect from Vorinostat (Fig 1A and 1C) and Ruxolitinib (Fig 1B and 1D) -induced apoptosis on SET-2 cells as direct contact with HS-5 cells (+ HS-5). Likewise, SET-2 cell viability in the presence of Vorinostat (Fig 1C) or Ruxolitinib (Fig 1D) was maintained by HS-5 cells even when the two cell types were cultured in transwell chambers that physically separated them, allowing only the exchange of soluble factors (+ HS-5 with TW). These results strongly suggest that HS-5 stromal cells protect MPN cells from apoptosis induced by Vorinostat and Ruxolitinib, at least in part, by means of soluble factors.

### Activation of JNK and PI3K signalling pathways is required for BM stromal–mediated protection of MPN cells

**Fig 2:**
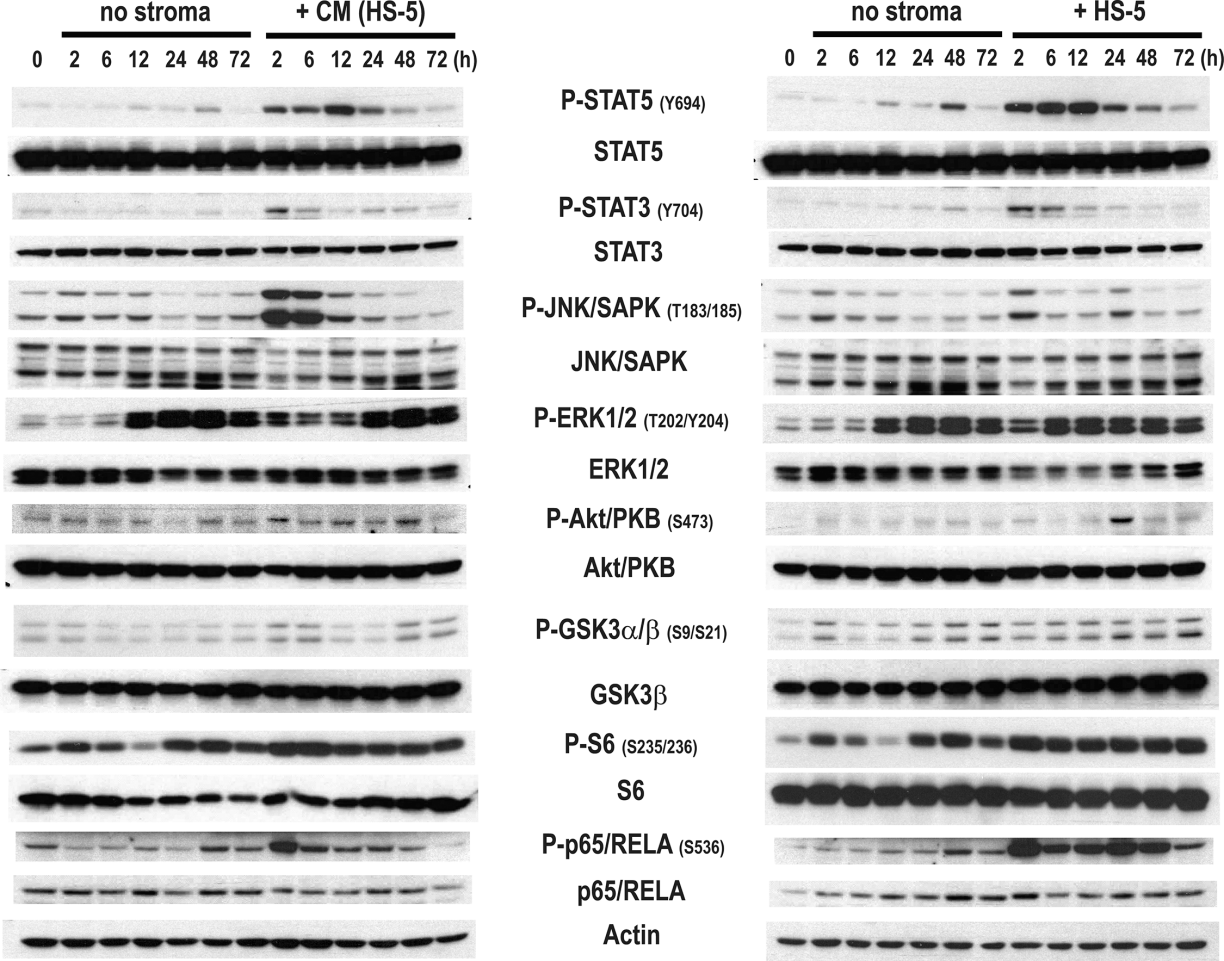
HS-5 stroma activates crucial signalling pathways in SET-2 cells. SET-2 cells were cultured *in vitro* (no stroma), co-cultured in a stromal layer of HS-5 cells (+ HS-5) and with HS-5 conditioned media [+ CM (HS-5)] at the indicated time point the cells. Cells were lysed and the phosphorylation and total levels of STAT5, STAT3, JNK/SAPK, ERK1/2, Akt/PKB, GSK3α/β, S6 and p65/RELA were analyzed by immunoblot. Actin was used as loading control. The data is representative of two independent experiments.

In order to understand the molecular mechanism by which the HS-5 protects MPN cells from drug-induced apoptosis, we analyzed the changes in activation of various signalling pathways induced by the system described above [35]. Signalling pathway activation was measured by assessing phosphorylation of downstream targets. We co-cultured SET-2 cells in the presence of a stromal layer of HS-5 cells (+ HS-5) and HS-5 conditioned media (+ CM) for a period of 72h and determined the activation of signalling pathways involved in cellular survival and proliferation by immunoblot analysis. As shown in Fig 2, the presence of HS-5 cells (+ HS-5) or their conditioned media (+ CM) activated the JAK-STAT, JNK, PI3K, NF-κB and, to a lesser extent, the MEK-ERK signalling pathways in a time-dependent manner. The kinetics of activation differed between the signalling pathways analyzed, and the direct contact between the cells or the presence of HS-5 conditioned media also induced differential activation patterns, as shown in the P-JNK/SAPK and P-Akt/PKB immunoblots (Fig 2). Interestingly, the activation of the signalling pathways is accompanied by an induction on the transcript levels of downstream target genes of the same pathways (data not shown) suggesting robust activation of these signalling pathways.

**Fig 3:**
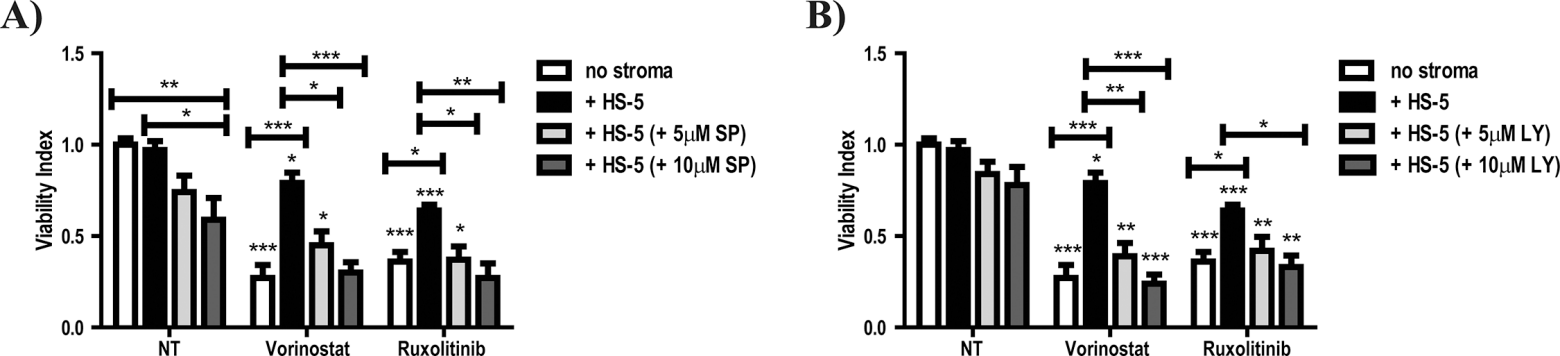
Pharmacological inhibition of JNK and PI3K signalling pathways completely reverted HS-5 BM stroma mediated protection of SET-2 cells. (A and B) SET-2 cells were cultured *in vitro* (no stroma) and co-cultured in a stromal layer of HS-5 cells (+ HS-5) for 72h in the presence of 2.0μM Vorinostat (Vor.), 500nM Ruxolitinib (Rux.), 5μM and 10μM SP600125 (JNK inhibitor) (in A) and 5μM and 10μM LY294002 (PI3K inhibitor) (in B). After 72h, the SET-2 cells were harvested, stained with CD45 (to distinguish between SET-2 and the stromal cell lines) and Annexin-V/PI or PI alone to determine cellular viability by Flow Cytometry analysis as described in “Materials and Methods”. The panels in (A and B) show the Viability Index graphs that normalize the viability values to those of the control condition (non-treated condition NT). Values indicate the mean ± standard deviation of the five experiments performed (* 0.05 >p; ** 0.01>p; *** 0.001>p).

We next tested the functional relevance of the observed pathway activation by treating SET-2 and HS-5 co-cultured cells with Vorinostat or Ruxolitinib in combination with pharmacological inhibitors of the pathways found to be activated: MEK/ERK (PD98059); NF-κB (BMS-345541); PI3K (LY294002) and JNK (SP600125). The co-treatment of SET-2 cells with Vorinostat or Ruxolinib with the MEK/ERK and NF-κB inhibitors had no impact on the HS-5 BM stroma mediated protection to SET-2 cells (data not shown). However, the treatment combination of either Vorinostat or Ruxolitinib with the JNK or PI3K pharmacological inhibitors completely reversed the HS-5 BM induced protection of SET-2 cells (Fig 3A and 3B). The decreased phosphorylation of JNK/SAPK protein following treatment with SP600125 and the decreased phosphorylation of PI3K downstream target GSK3α/β following treatment with LY294002 (S5 Fig) indicate these drugs are indeed inhibiting these signalling pathways in our co-culture system.

### Combined treatment of Vorinostat and Ruxolitinib with JNK and PI3K inhibition results in synergistic induction of cell death in MPN cells

Given that the inhibition of both JNK and PI3K signalling pathways completely reversed the HS-5 BM induced protection of SET-2 cells, we tested whether the combination of the different pharmacological agents could have synergist effects using the Chou-Talalay method [32]. To this end, we cultured SET-2 for 72h with or without the HS-5 cells and treated with escalating concentrations of the combinations of Vorinostat and Ruxolitinib with both SP600125 (JNKi) and LY294002 (PI3Ki). As shown in S6 Fig, the protective effect of the HS-5 increased the EC50 of both Vorinostat and Ruxolitinib in SET-2 cells by almost 3 fold (2.19 for Vorinostat and 2.60 for Ruxolitinib). Combining both SP600125 (JNKi) and LY294002 (PI3Ki) with Vorinostat (S6A and S6B Fig) reduced the EC50 (3.30μM to 0.46μM–JNKi and 3.30μM to 1.41μM–PI3Ki) further demonstrating that the inhibition of these pathways abrogated the protective effect induced by the HS-5. Furthermore, the combination indexes (CI) of SET-2 cells co-cultured with HS-5 cells and exposed to Vorinostat and SP600125 (JNKi) or LY294002 (PI3Ki) showed a synergistic effect (CI below 1.00) between the drugs at therapeutically achievable doses of Vorinostat (X < 2.52μM) [42]. On the other hand, the combination of SP600125 (JNKi) with Ruxolitinib reduced the EC50 (0.26μM to 0.19μM) but not the combination of LY294002 (PI3Ki) with Ruxolitinib (0.26μM to 0.31μM) (S6C and S6D Fig). However, both drug combinations induce higher degree of cell death and these effects are synergistic at therapeutically achievable doses of Ruxolitinib (X < 1.00μM) [43]. These results further demonstrate that the HS-5 cells activate JNK and PI3K signalling pathways in SET-2 cells, probably mediating resistance to apoptosis induced by Vorinostat and Ruxolitinib.

### JNK and PI3K signaling pathway activation mediates *ex-vivo* stromal protection of primary MPN samples

**Fig 4:**
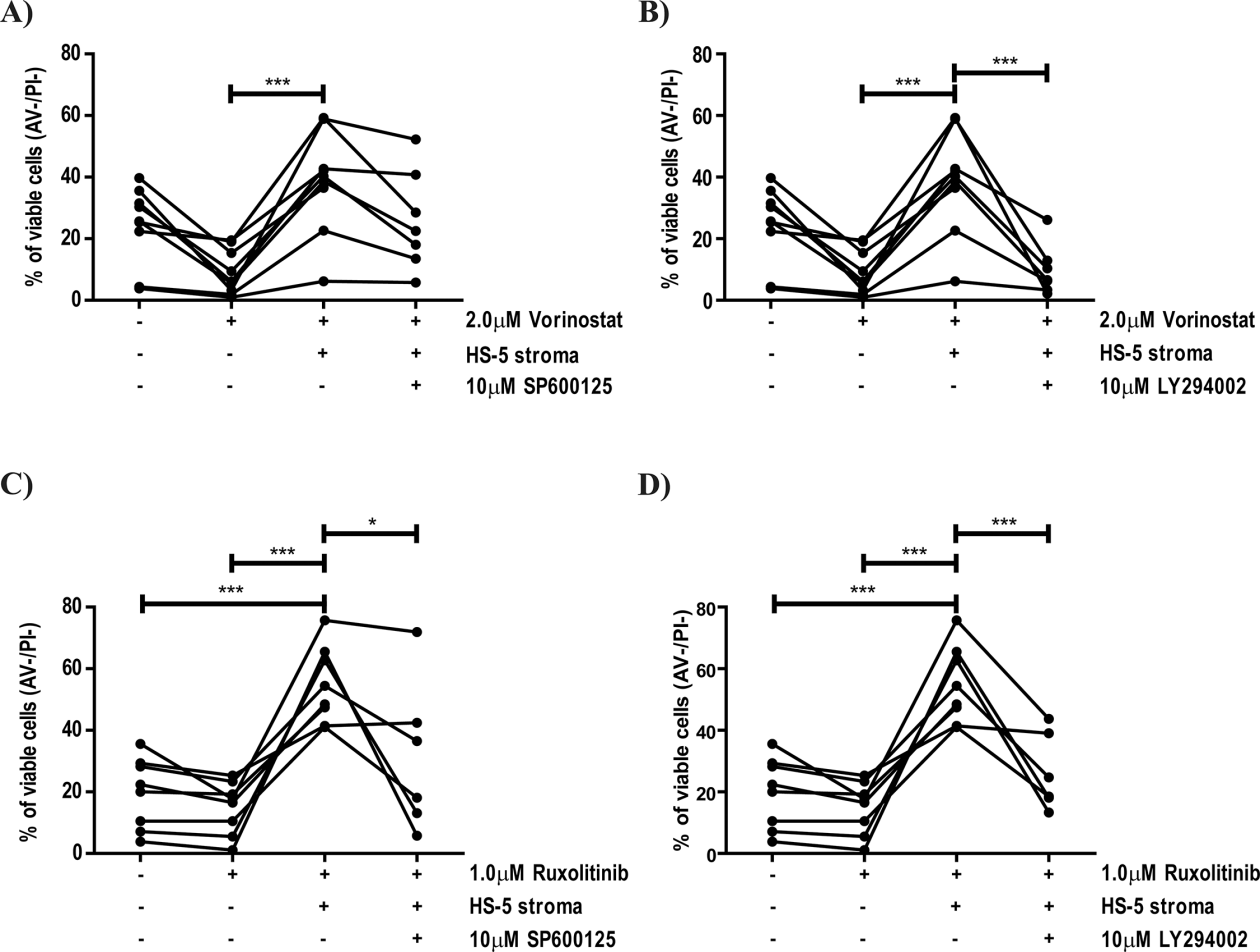
Pharmacological inhibition of JNK and PI3K signalling pathways abrogate the HS-5 BM stroma induced protection of patient derived BM Lin^-^CD34^+^ cells from Vorinostat–induced cytotoxicity. Lin^-^CD34^+^ cells were isolated as described in “Material and Methods” section and co-cultured in a stromal layer of HS-5 cells for 72h in presence of the indicated concentrations of Vorinostat (A and B), Ruxolitinib (C and D) SP600125 (JNKi–A and C) and LY294002 (PI3Ki–B and D). At 72h of co-culture, cells were harvested, stained with CD45 (to distinguish between Lin^-^CD34^+^ and HS-5 cells) and Annexin-V/PI to determine cellular viability by Flow Cytometry analysis as described in “Materials and Methods”. The results are representative of nine MPN patients tested and graphics indicate the percentage of viable cells (Annexin-V and PI negative cells) in the indicated conditions (* 0.05 >p; *** 0.001>p).

The above findings were confirmed in primary Lin^-^CD34^+^ cells from MPN patients (Table 1), as with the cell lines, these were co-cultured with HS-5 BM stromal cells in the presence of the pharmacological agents. Both Vorinostat (Fig 4A and 4B) and Ruxolitinib (Fig 4C and 4D) reduced cellular viability, although the degree of reduction varied between patients (Table 1). Furthermore, co-culture with HS-5 cells significantly protected primary cells from Vorinostat- (Fig 4A and 4B) and Ruxolitinib- (Fig 4C and 4D) induced apoptosis. Inhibition of JNK (Fig 4A and 4C) and PI3K (Fig 4B and 4D) signalling pathways reversed the protective effect of HS-5 cells, although the reversion was not as striking as with the cell line model (Fig 3) and was not seen in all the MPN patient samples (MPN #1; #3 and #6). Nonetheless, the results obtained with MPN primary samples support our hypothesis that the stroma-mediated protection of MPN cells from drug-induced apoptosis requires the activation of JNK and PI3K signalling pathways.

## Discussion

The unraveling of the role of JAK2 activation in the molecular pathophysiology of MPN [4–7] led to the development of JAK2 inhibitors, such as Ruxolitinib. Despite clear efficacy in reducing disease burden, these agents have failed to eradicate the malignant clone [11, 12]. Similarly, in light of the identification of epigenetic alterations in MPN, HDAC inhibitors have been tested in these diseases [13–15]. These yielded modest responses and poor tolerability, but Vorinostat was successful in reducing tumour burden in some patients [14]. We postulated that the BM microenvironment could play a role in protecting the MPN cells from the cytotoxic actions of Ruxolitinib and Vorinostat. There is increasing evidence implicating the BM microenvironment in the resistance to therapy in haematological malignancies. Tesfai and colleagues reported that the BM stroma impaired the response of B-cell Acute Lymphoblatic Leukemia (B-ALL) cells to cytotoxic agents [23]. In myeloid neoplasms, it was recently reported that tyrosine kinase inhibitors are unable to completely eliminate the leukaemic stem cell in Chronic Myeloid Leukemia cells due to microenvironmental cues [24, 26]. Similarly, a variety of agents fail to kill Acute Myeloid Leukemia cells [25, 44, 45] in the presence of BM stromal cells. Here, we demonstrate that the stromal cells protect MPN derived cell lines and primary MPN Lin^-^ CD34^+^ cells from apoptosis induced by Vorinostat and Ruxolitinib (Figs 1 and 4).

Several studies indicate that the BM stroma secretes cytokines which protect neoplastic myeloid cells from drug-induced apoptosis [24, 25, 27]. Our studies indicate that soluble factors produced by the stromal cell line HS-5 protected MPN cells from Vorinostat- and Ruxolitinib- induced apoptosis (Fig 1). Analysis of activation of downstream signalling pathways indicated that these factors activate several signalling pathways (Fig 2) in MPN cells. We have not yet identified the secreted factor responsible for this effect but our data indicate that it probably acts through JNK and PI3K signalling pathway activation. Consistent with this is the preliminary data from our laboratory suggesting that SCF and GM-CSF could be implicated in the protection (data not shown). These cytokines are reported to activate MEK-ERK, NF-κB and PI3K signalling pathways [46, 47], and it is tempting to speculate that they might activate these pathways in our cell-culture system (Fig 2). The activation of JNK signalling pathway by the secreted factors might occur through IL1β signalling, a cytokine associated with inflammation [48, 49] and also secreted by HS-5 cells [35]. The identification of the secreted factor(s) responsible for the protective effect will be important in delineating possible therapeutic targets.

Despite the fact that our data implicate the role of a HS-5 secreted factor in the protective effect, the importance of the direct contact (or juxtacrine effect) between stromal and MPN cells cannot be ignored. Higher levels of MPN cellular viability were obtained when MPN cells were in direct contact with the HS-5 BM stroma (Fig 1), particularly when treated with Ruxolitinib (Fig 1B). These results might suggest that a combination of juxtacrine and paracrine effects of the HS-5 BM stroma could contribute to the protection of MPN cells. This is further reinforced by the fact that adhesion molecules, such as ICAM-1 and E-Cadherin, have been reported to activate the signalling pathways that we observed in our co-culture system (Fig 2) [50, 51].

In an attempt to improve the efficacy of JAK2 inhibitors, several clinical trials in the MPN field are testing combinations of Ruxolitinib with drugs which may have synergistic mechanisms of action, such as signalling inhibitors, immunomodulators and epigenetic modulators [52, 53]. Given our results showing the BM stroma mediated activation of JNK and PI3K signalling pathways in MPN cells (Fig 2), we tested combinations of Vorinostat and Ruxolitinib with pharmacological inhibitors of such pathways. In these combinations, JNK (SP600125) and PI3K (LY294002) inhibitors, abrogated the protective effects of stromal cells on MPN cells in the cell line model (Fig 3) and also in primary BM-derived Lin^-^CD34^+^ MPN cells (Fig 4), demonstrating that the integrity of these signalling pathways must be maintained in order for the HS-5 cells to exert their protective effect. This is supported by the fact that both JNK and PI3K signalling pathways have been implicated in the regulation of cellular viability in haematological malignancies [54–56]. Furthermore, our results (Figs 3 and 4) are agreement with the fact that the PI3K signalling pathway has been implicated in the protection of myeloid neoplastic cells to several cytotoxic stimuli [45, 57], suggesting that the PI3K axis may act as a gatekeeper of the BM mediated protection in myeloid neoplasms. As shown in Fig 4, the level of reversion of the HS-5-induced protection to Vorinostat and Ruxolitinib with SP600125 and LY294002 varied among primary MPN cells (MPN patients #1, #3 and #6 failed to revert HS-5 induced protection with SP600125 and LY294002), which might reflect inter-patient variability and might also suggest that other signalling pathways could be activated in these patients by stromal cells, such as MEK-ERK or NF-κB (Fig 2). These results suggest the possibility of a novel therapeutic approach for the treatment of MPN and other haematological malignancies: the dual targeting of the neoplastic clone (MPN cells) and the microenvironmental cues (stromal-activated signalling pathways). This therapeutic strategy has the potential to overcome stromal-induced drug resistance and expose the neoplastic stem cells to eradication. Importantly, the combination of Ruxolitinib and BKM120 (a pan-PI3K inhibitor) is already being tested in a phase I clinical in patients with myelofibrosis (NCT01730248), despite the fact that the study trial was designed with a different propose.

**Fig 5:**
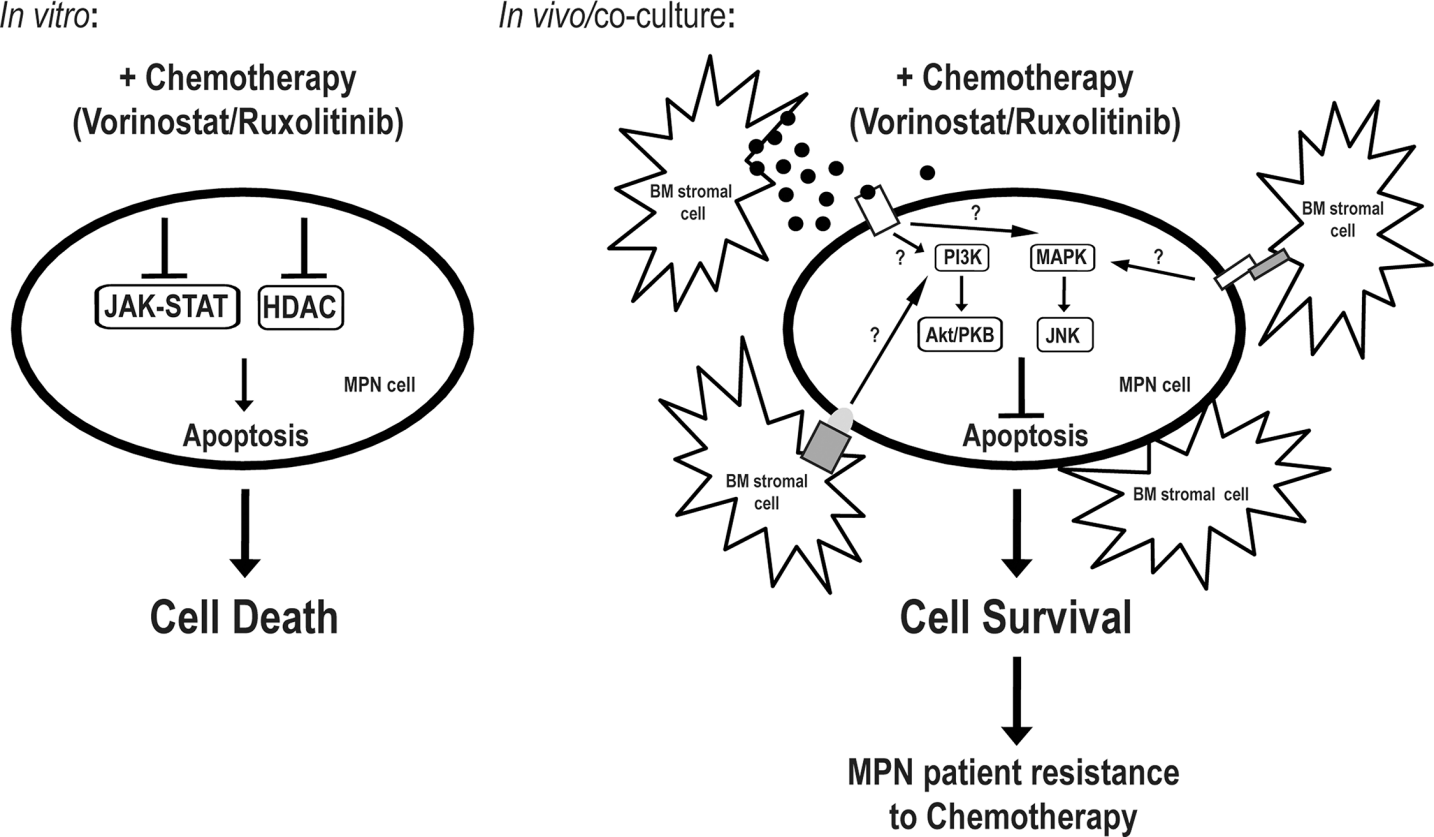
Model for the proposed BM mediated protection of MPN cells. *In vitro*, MPN cells (SET-2, HEL, UKE-1 cell lines and MPN patient BM-derived Lin^-^CD34^+^ cells) are killed once exposed to chemotherapeutic agents, like Vorinostat and Ruxolitinib, as a result of the inhibition of HDAC enzymes and JAK2V617F mutant protein kinase, respectively. However, *in vivo* (in MPN patients) or in the co-culture assays with the HS-5 BM stroma, MPN cells are able survive the cytotoxic action of these noxious agents through the activation of the JNK and PI3K signalling pathways, allowing MPN cells to escape these chemotherapeutic regiments and prolonging disease in MPN patients. The mechanisms underlying the activation of these signalling pathways are still elusive, but we postulate that involves the secretion of soluble factors by the BM stroma and the direct interaction between the MPN cells and the BM microenvironment.

In summary, we have demonstrated that the BM stroma plays a pivotal role in the protection of MPN cells from apoptosis induced by Vorinostat and Ruxolitinib. In the absence of stromal cells and/or their secreted factors, Vorinostat and Ruxolitinib lead to MPN cell death. However, when MPN cells are placed in contact with BM stromal cells, this leads to the activation of JNK and the PI3K signalling pathways which protect MPN cells from Vorinostat and Ruxolitinib induced apoptosis (Fig 5). Inhibition of these pathways in the same system abrogates the protective effect exerted by the stroma. These results point to a novel potential therapeutic approach to treat MPN patients: the dual targeting of both the neoplastic clone and the microenvironmental cues.

## Supporting Information

S1 Fig HS-5 stromal cells protect MPN cells from Vorinostat and Ruxolitinib- induced apoptosis in a time-dependent manner. SET-2 cells were cultured *in vitro* (no stroma) and co-cultured with a stromal layer of HS-5 cells (+ HS-5) up to 144h and treated with the indicated concentrations of Vorinostat (A) and Ruxolitinib (B). At the indicated time points, SET-2 cells were harvested, stained with CD45 (to distinguish between SET-2 and the stromal cell lines) and Annexin-V/PI or PI alone to determine cellular viability by Flow Cytometry analysis as described in the “Material and Methods” section. The panels show the Viability Index graphs that normalize the viability values to those of the 0h time point. Values indicate the mean ± standard deviation of the three experiments performed (* 0.05 >p; ** 0.01>p; *** 0.001 > p).
(TIF)

S2 Fig Vorinostat and Ruxolitinib modulate expression of HDAC and JAK-STAT pathways responsive genes. SET-2 cells were cultured *in vitro* (no stroma) and co-cultured in a stromal layer of HS-5 cells (+ HS-5) for 24h in the presence of 2.0μM Vorinostat (A) and 500nM Ruxolitinib (B). The transcript levels of the indicated genes (A–*CDKN1A*; *IER3* and *BIRC3* / B–*BCL2*; *OSM* and *PIM1*) were evaluated as described in the “Material and Methods” section. The values of each gene were normalized to *HPRT1* and depicted as relative values of the control condition (no stroma–A—0.0μM Vorinostat and B – 0nM Ruxolitinib). Values indicate the mean ± standard deviation of duplicates (* 0.05 >p; ** 0.01>p; *** 0.001>p).
(TIF)

S3 Fig HS-5 stromal cells protect MPN cells from Vorinostat and Ruxolitinib- induced apoptosis. HEL (A) and UKE-1 (B) cells were cultured *in vitro* (no stroma) and co-cultured with a stromal layer of HS-5 cells (+ HS-5) for 72h and incubated with the indicated concentrations of Vorinostat and Ruxolitinib. At 72h of co-culture, HEL and UKE-1 cells were harvested, stained with CD45 (to distinguish between MPN cells and the HS-5 stromal cell line) and Annexin-V/PI or PI alone to determine cellular viability by Flow Cytometry analysis as described in the “Material and Methods” section. The panels show the Viability Index graphs that normalize the viability values to the viability values of the control conditions (0.0μM Vorinostat and 0.0μM Ruxolitinib). Values indicate the mean ± standard deviation of triplicates (A) and quadriplicates (B) (* 0.05>p; ** 0.01>p; *** 0.001>p).
(TIF)

S4 Fig HS-5 and KM-102 stromal cells protect SET-2 cells from Vorinostat and Ruxolitinib- induced apoptosis. SET-2 cells were cultured *in vitro* (no stroma) and co-cultured with a stromal layer of HS-5 cells (+ HS-5) and KM-102 cells (+ KM-102) for 72h and incubated with the indicated concentrations of Vorinostat (A) and Ruxolitinib (B). At 72h of co-culture, SET-2 cells were harvested, stained with CD45 (to distinguish between SET-2 and the stromal cell lines) and Annexin-V/PI or PI alone to determine cellular viability by Flow Cytometry analysis as described in the “Material and Methods” section. The A and B panels show the Viability Index graphs that normalize the viability values to the viability values of the control conditions (A—0.0μM Vorinostat and B—0nM Ruxolitinib). Values indicate the mean ± standard deviation of triplicates (* 0.05>p; ** 0.01>p; *** 0.001>p).
(TIF)

S5 Fig Pharmacological inhibition of JNK and PI3K decreases phosphorylation of downstream modulators of signalling pathways. SET-2 cells were cultured *in vitro* (no stroma), co-cultured in a stromal layer of HS-5 cells (+ HS-5) and with HS-5 conditioned media [+ CM (HS-5)] with or without 10μM SP600125 and 10μM LY294002 for 24h. Cells were lysed and the phosphorylation and total levels of STAT5, STAT3, JNK/SAPK and GSK3α/β were analyzed by immunoblot. Actin was used as loading control. The data is representative of two independent experiments.
(TIF)

S6 Fig Pharmacological inhibition of JNK and PI3K synergistically interacts with Vorinostat and Ruxolitinib to revert HS-5 stroma mediated protection of SET-2 cells. SET-2 cells were cultured *in vitro* (no stroma) and co-cultured in a stromal layer of HS-5 cells (+ HS-5) for 72h with increasing concentrations of Vorinostat (A and B) and Ruxolitinib (C and D) (10 concentrations ranging from 0.0 to 8.0μM) that were combined with increasing doses of SP600125 (A and C) and LY294002 (B and D) (10 concentrations ranging from 0.0 to 80μM). At 72h of co-culture, SET-2 cells were harvested, stained with CD45 (to distinguish between SET-2 and the stromal cell lines) and PI to determine cellular viability by Flow Cytometry analysis as described in the “Material and Methods” section. The graphs in the panels show the dose response curves of the drugs in the following conditions: no stroma; + HS-5 and + HS-5 + Drug (SP or LY). The EC50 and the Combination Indexes for each of the drug combinations are show and were calculated as described in “Materials and Methods” section. The data is representative of three independent experiments.
(TIF)

S1 Table Drug concentrations used to calculated EC50 and drug interaction. (DOCX)

S2 Table Primers used in the quantitative-RealTime-PCR (qPCR). (DOCX)

S3 Table Antibodies used in the Immunoblotting. (DOCX)
