# Supplementary figures and images for: The Bone Marrow-Mediated Protection of Myeloproliferative Neoplastic Cells to Vorinostat and Ruxolitinib Relies on the Activation of JNK and PI3K Signalling Pathways
Source: PLoS One. 2015 Dec 1;10(12):e0143897. doi: 10.1371/journal.pone.0143897 (PMC4666616; doi:10.1371/journal.pone.0143897)

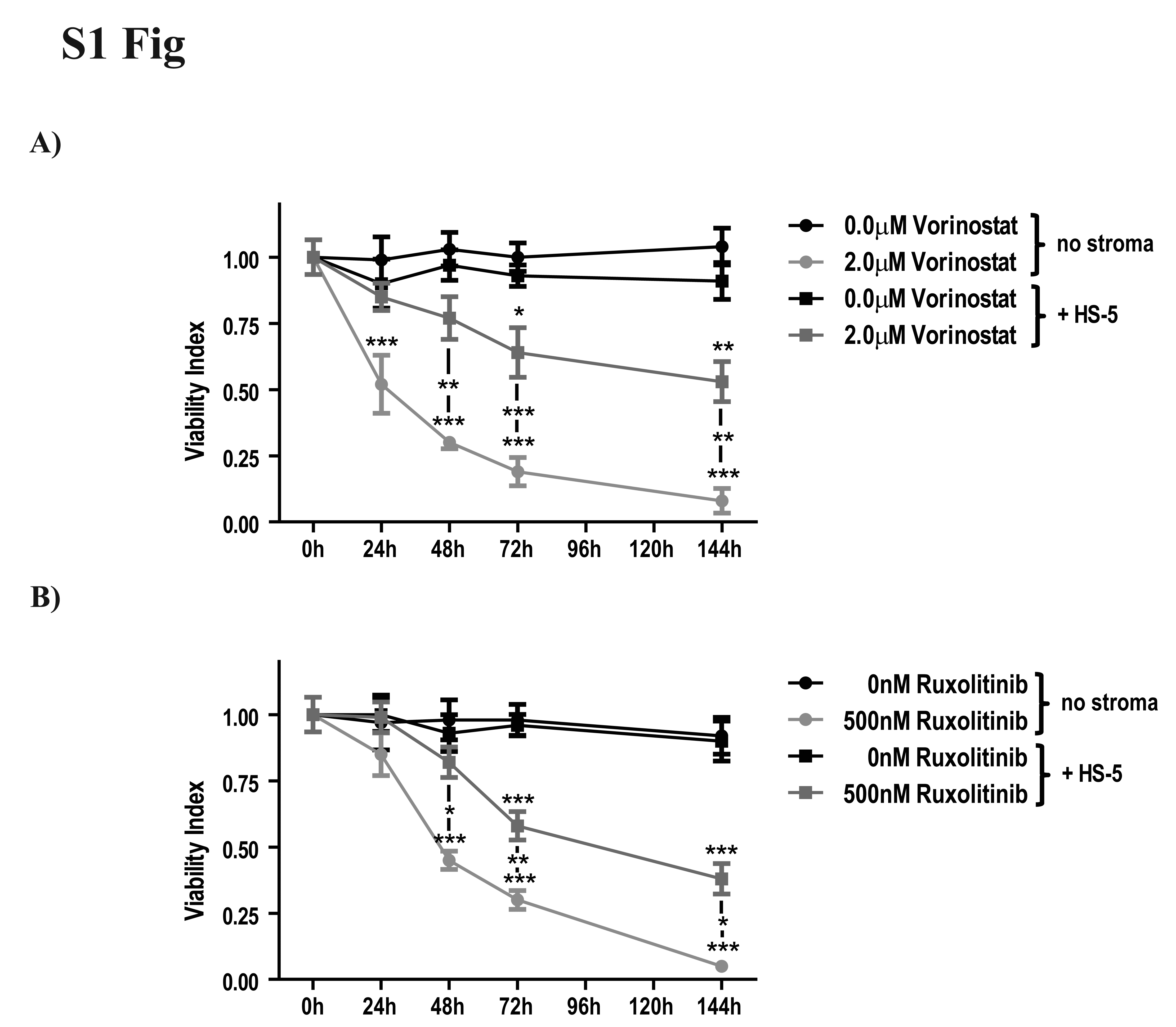

Supplement: S1 Fig — SET-2 cells were cultured in vitro (no stroma) and co-cultured with a stromal layer of HS-5 cells (+ HS-5) up to 144h and treated with the indicated concentrations of Vorinostat (A) and Ruxolitinib (B). At the indicated time points, SET-2 cells were harvested, stained with CD45 (to distinguish between SET-2 and the stromal cell lines) and Annexin-V/PI or PI alone to determine cellular viability by Flow Cytometry analysis as described in the “Material and Methods” section. The panels show the Viability Index graphs that normalize the viability values to those of the 0h time point. Values indicate the mean ± standard deviation of the three experiments performed (* 0.05 >p; ** 0.01>p; *** 0.001 > p). (TIF) [file pone.0143897.s001.tif]

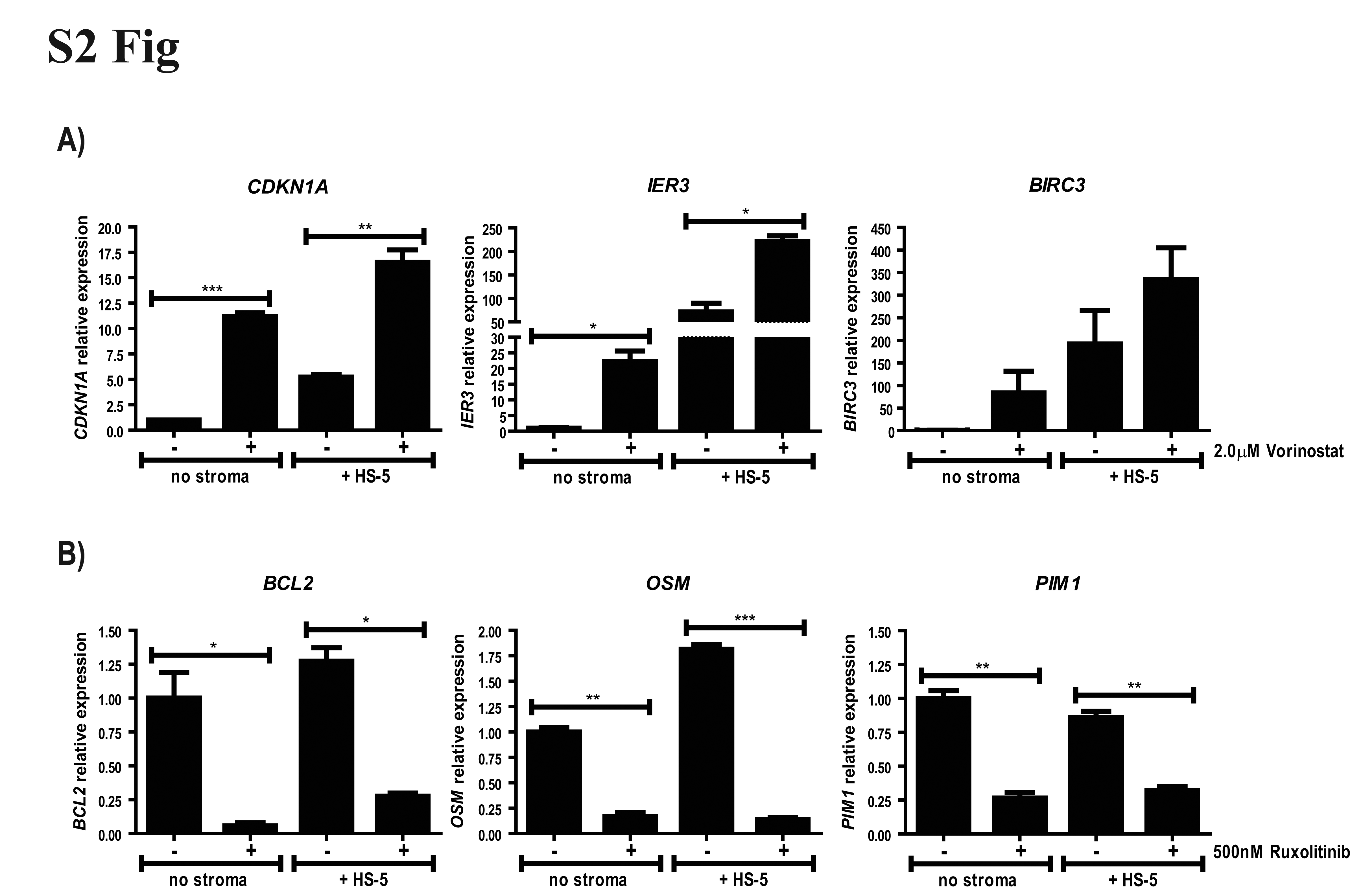

Supplement: S2 Fig — SET-2 cells were cultured in vitro (no stroma) and co-cultured in a stromal layer of HS-5 cells (+ HS-5) for 24h in the presence of 2.0μM Vorinostat (A) and 500nM Ruxolitinib (B). The transcript levels of the indicated genes (A–CDKN1A; IER3 and BIRC3 / B–BCL2; OSM and PIM1) were evaluated as described in the “Material and Methods” section. The values of each gene were normalized to HPRT1 and depicted as relative values of the control condition (no stroma–A—0.0μM Vorinostat and B – 0nM Ruxolitinib). Values indicate the mean ± standard deviation of duplicates (* 0.05 >p; ** 0.01>p; *** 0.001>p). (TIF) [file pone.0143897.s002.tif]

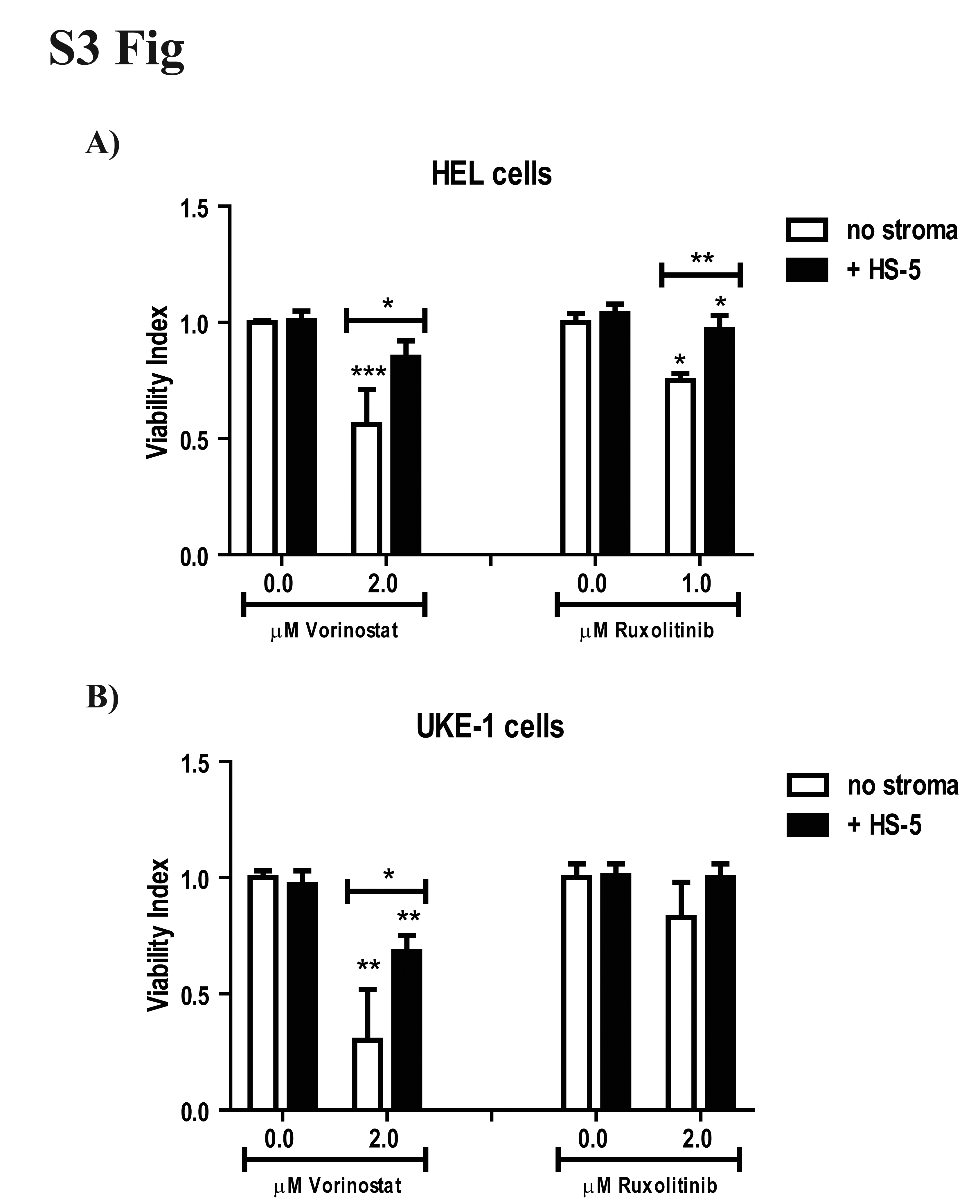

Supplement: S3 Fig — HEL (A) and UKE-1 (B) cells were cultured in vitro (no stroma) and co-cultured with a stromal layer of HS-5 cells (+ HS-5) for 72h and incubated with the indicated concentrations of Vorinostat and Ruxolitinib. At 72h of co-culture, HEL and UKE-1 cells were harvested, stained with CD45 (to distinguish between MPN cells and the HS-5 stromal cell line) and Annexin-V/PI or PI alone to determine cellular viability by Flow Cytometry analysis as described in the “Material and Methods” section. The panels show the Viability Index graphs that normalize the viability values to the viability values of the control conditions (0.0μM Vorinostat and 0.0μM Ruxolitinib). Values indicate the mean ± standard deviation of triplicates (A) and quadriplicates (B) (* 0.05>p; ** 0.01>p; *** 0.001>p). (TIF) [file pone.0143897.s003.tif]

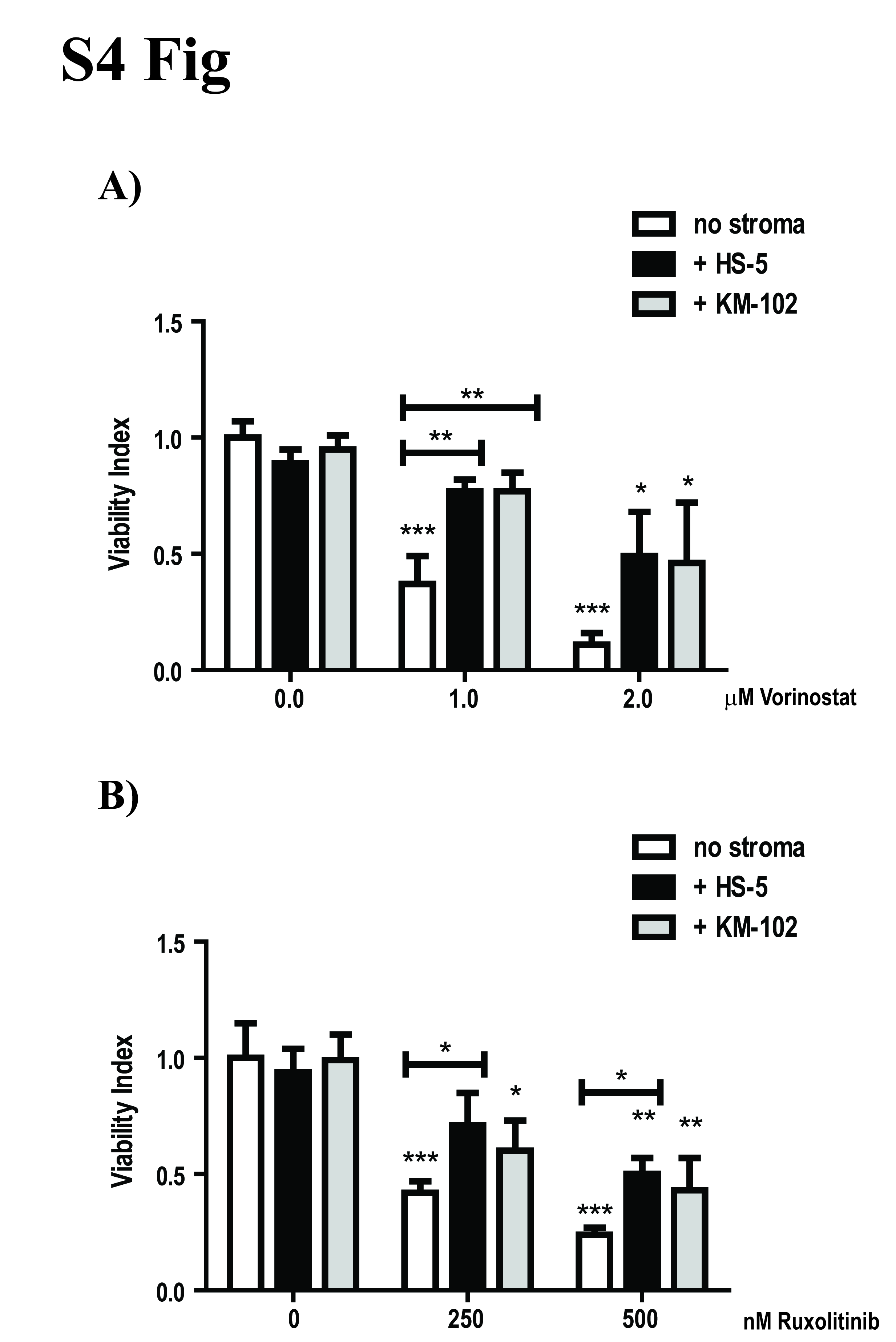

Supplement: S4 Fig — SET-2 cells were cultured in vitro (no stroma) and co-cultured with a stromal layer of HS-5 cells (+ HS-5) and KM-102 cells (+ KM-102) for 72h and incubated with the indicated concentrations of Vorinostat (A) and Ruxolitinib (B). At 72h of co-culture, SET-2 cells were harvested, stained with CD45 (to distinguish between SET-2 and the stromal cell lines) and Annexin-V/PI or PI alone to determine cellular viability by Flow Cytometry analysis as described in the “Material and Methods” section. The A and B panels show the Viability Index graphs that normalize the viability values to the viability values of the control conditions (A—0.0μM Vorinostat and B—0nM Ruxolitinib). Values indicate the mean ± standard deviation of triplicates (* 0.05>p; ** 0.01>p; *** 0.001>p). (TIF) [file pone.0143897.s004.tif]

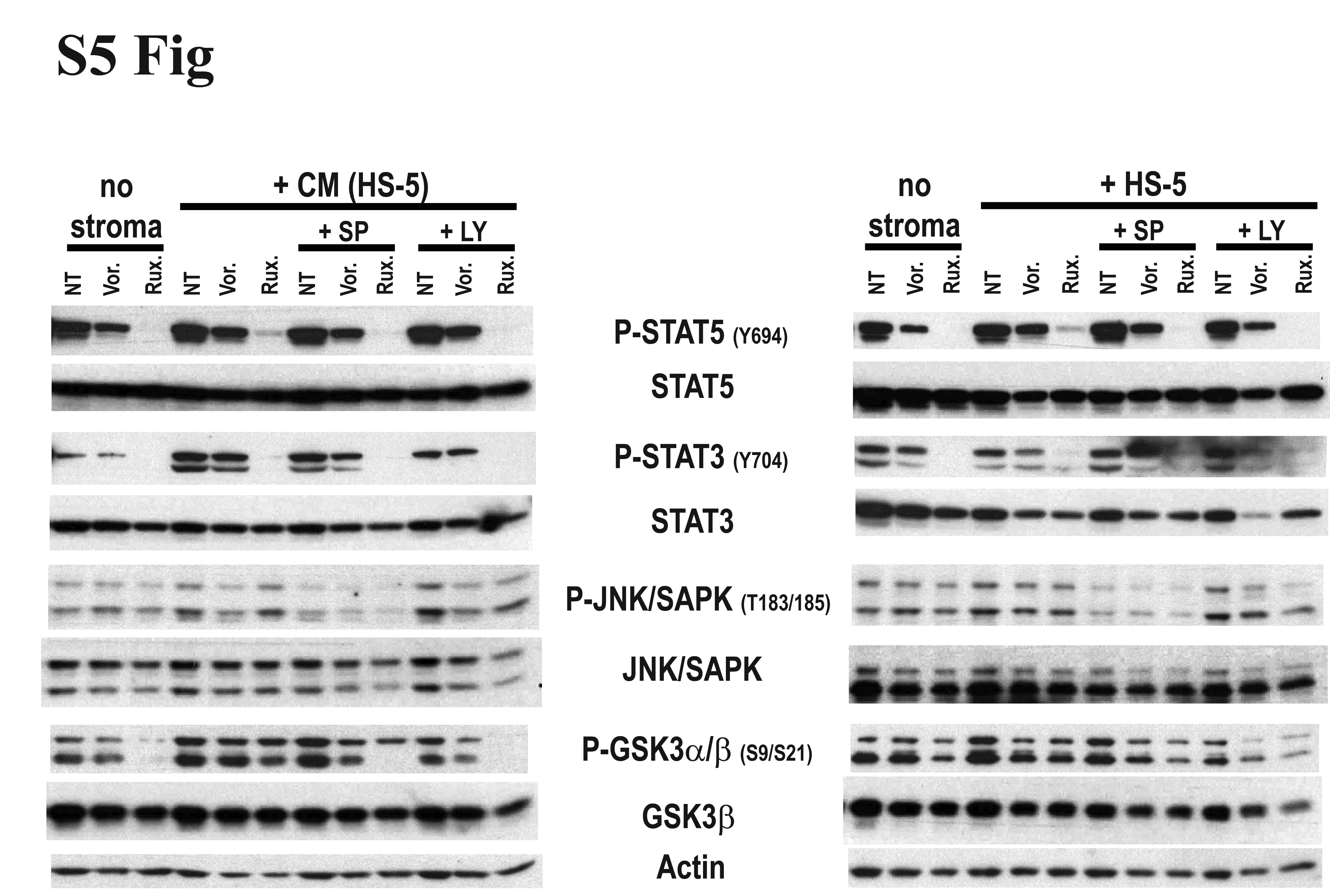

Supplement: S5 Fig — SET-2 cells were cultured in vitro (no stroma), co-cultured in a stromal layer of HS-5 cells (+ HS-5) and with HS-5 conditioned media [+ CM (HS-5)] with or without 10μM SP600125 and 10μM LY294002 for 24h. Cells were lysed and the phosphorylation and total levels of STAT5, STAT3, JNK/SAPK and GSK3α/β were analyzed by immunoblot. Actin was used as loading control. The data is representative of two independent experiments. (TIF) [file pone.0143897.s005.tif]

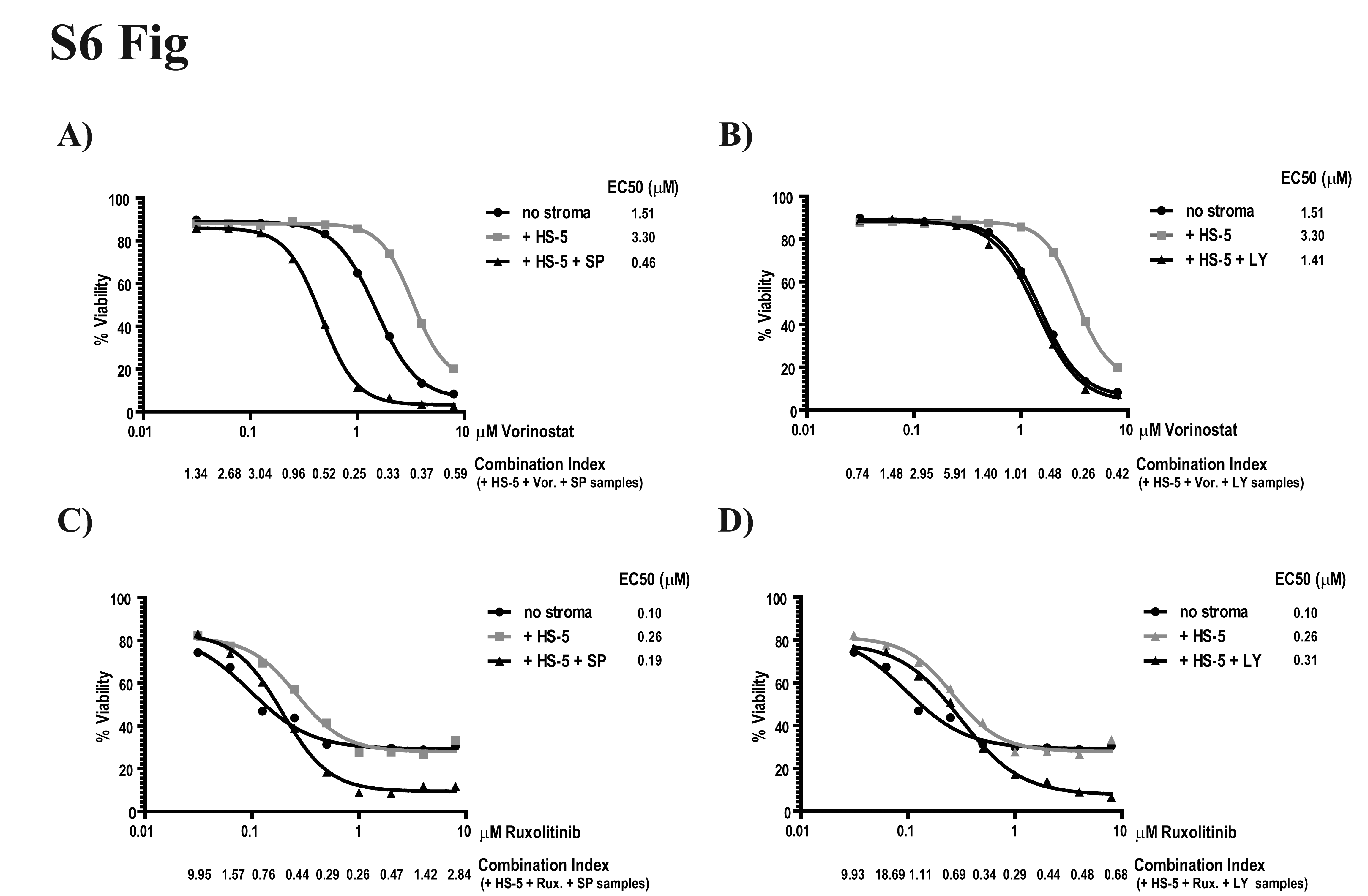

Supplement: S6 Fig — SET-2 cells were cultured in vitro (no stroma) and co-cultured in a stromal layer of HS-5 cells (+ HS-5) for 72h with increasing concentrations of Vorinostat (A and B) and Ruxolitinib (C and D) (10 concentrations ranging from 0.0 to 8.0μM) that were combined with increasing doses of SP600125 (A and C) and LY294002 (B and D) (10 concentrations ranging from 0.0 to 80μM). At 72h of co-culture, SET-2 cells were harvested, stained with CD45 (to distinguish between SET-2 and the stromal cell lines) and PI to determine cellular viability by Flow Cytometry analysis as described in the “Material and Methods” section. The graphs in the panels show the dose response curves of the drugs in the following conditions: no stroma; + HS-5 and + HS-5 + Drug (SP or LY). The EC50 and the Combination Indexes for each of the drug combinations are show and were calculated as described in “Materials and Methods” section. The data is representative of three independent experiments. (TIF) [file pone.0143897.s006.tif]
