# Supplementary material for: The Bone Marrow-Mediated Protection of Myeloproliferative Neoplastic Cells to Vorinostat and Ruxolitinib Relies on the Activation of JNK and PI3K Signalling Pathways
Source: PLoS One. 2015 Dec 1;10(12):e0143897. doi: 10.1371/journal.pone.0143897 (PMC4666616; doi:10.1371/journal.pone.0143897)
Supplement: S1 Table — (DOCX) [file pone.0143897.s007.docx]

**S1 Table.** Drug concentrations used to calculated EC50 and drug interaction.

| **#1** | **#2** | **#3** | **#4** |
| --- | --- | --- | --- |
| **Vorinostat**  **(µM)** | **Ruxolitinib**  **(µM)** | **JNKi-SP600125**  **(µM)** | **PI3Ki-LY294002 (µM)** |
| 0.0 | 0.0 | 0.0 | 0.0 |
| 0.03125 | 0.03125 | 0.3125 | 0.3125 |
| 0.0625 | 0.0625 | 0.625 | 0.625 |
| 0.125 | 0.125 | 1.25 | 1.25 |
| 0.25 | 0.25 | 2.5 | 2.5 |
| 0.5 | 0.5 | 5.0 | 5.0 |
| 1.0 | 1.0 | 10.0 | 10.0 |
| 2.0 | 2.0 | 20.0 | 20.0 |
| 4.0 | 4.0 | 40.0 | 40.0 |
| 8.0 | 8.0 | 80.0 | 80.0 |
