# Supplementary material for: The Bone Marrow-Mediated Protection of Myeloproliferative Neoplastic Cells to Vorinostat and Ruxolitinib Relies on the Activation of JNK and PI3K Signalling Pathways
Source: PLoS One. 2015 Dec 1;10(12):e0143897. doi: 10.1371/journal.pone.0143897 (PMC4666616; doi:10.1371/journal.pone.0143897)
Supplement: S2 Table — (DOCX) [file pone.0143897.s008.docx]

**S2 Table.** Primers used in the quantitative-Real-Time-PCR (qPCR).

| **Gene** | **Forward primer 5'🡪 3'** | **Reverse primer 5' 🡪 3'** |
| --- | --- | --- |
| ***BCL2*** | ATGTGTGTGGAGAGCGTCAACC | TGAGCAGAGTCTTCAGAGACAGCC |
| ***BIRC3*** | ACACATGCAGCCCGCTTTA | CTCCAGATTCCCAACACCTGA |
| ***CDKN1A*** | GAGACTCTCAGGGTCGAAAACG | ATTAGGGCTTCCTCTTGGAGAAG |
| ***HPRT1*** | TGACACTGGCAAAACAATGCA | GGTCCTTTTCACCAGCAAGCT |
| ***IER3*** | AACCGAACCCAGCCAAAAG | CCATCAGGATCTGGCAGAAGA |
| ***OSM*** | CACAGACTGGCCGACTTAGAG | AGTCCTCGATGTTCAGCCCA |
| ***PIM1*** | CGAGCATGACGAAGAGATCAT | TCGAAGGTTGGCCTATCTGA |
