# Supplementary material for: The Bone Marrow-Mediated Protection of Myeloproliferative Neoplastic Cells to Vorinostat and Ruxolitinib Relies on the Activation of JNK and PI3K Signalling Pathways
Source: PLoS One. 2015 Dec 1;10(12):e0143897. doi: 10.1371/journal.pone.0143897 (PMC4666616; doi:10.1371/journal.pone.0143897)
Supplement: S3 Table — (DOCX) [file pone.0143897.s009.docx]

**S3 Table.** Antibodies used in the immunoblotting.

| **Protein** | **Host** | **Manufacturer** |
| --- | --- | --- |
| **Actin** | Goat | Santa Cruz Biotechnology |
| **Akt/PKB** | Rabbit | Cell Signaling Technology |
| **ERK1/2** | Rabbit | Cell Signaling Technology |
| **GSK3β** | Rabbit | Cell Signaling Technology |
| **JNK/SAPK** | Rabbit | Cell Signaling Technology |
| **P-Akt/PKB (S473)** | Rabbit | Cell Signaling Technology |
| **P-ERK1/2 (T202/Y204)** | Rabbit | Cell Signaling Technology |
| **P- GSK3α/β (S9/S21)** | Rabbit | Cell Signaling Technology |
| **P-JNK/SAPK (T183/185)** | Rabbit | Cell Signaling Technology |
| **P65/RELA** | Rabbit | Cell Signaling Technology |
| **P-p65/RELA (S536)** | Rabbit | Cell Signaling Technology |
| **P-S6 (S235/236)** | Rabbit | Cell Signaling Technology |
| **P-STAT3 (Y05)** | Mouse | Cell Signaling Technology |
| **P-STAT5A/B (Y694)** | Rabbit | Cell Signaling Technology |
| **S6** | Rabbit | Cell Signaling Technology |
| **STAT3** | Rabbit | Cell Signaling Technology |
| **STAT5A/B** | Mouse | Santa Cruz Biotechnology |
